# Background Tissue with Native Target Expression Can Determine Presence of Nodal Metastasis in Head and Neck Squamous Cell Carcinoma Patients Infused with Targeted Fluorescent Tracers

**DOI:** 10.1007/s11307-025-01996-4

**Published:** 2025-03-18

**Authors:** Nicole Meeks, Sherin James, Giri Krishnan, Akhilesh Wodeyar, Hidenori Tanaka, Benjamin B. Kasten, Yu-Jin Lee, Marisa E. Hom, Eben L. Rosenthal, Jason M. Warram

**Affiliations:** 1 Department of Otolaryngology-Head and Neck Surgery, Vanderbilt University Medical Center, Nashville, TN USA; 2 Department of Otolaryngology, The University of Alabama at Birmingham, Birmingham, AL USA; 3 Department of Otolaryngology, The University of Adelaide, Adelaide, South Australia Australia; 4 Department of Otolaryngology-Head and Neck Surgery, Stanford University School of Medicine, Stanford, CA USA

**Keywords:** Squamous cell cancer, Lymph node, Anti-EGFR antibody, Intraoperative molecular imaging, Fluorescent guided surgery, Cancer surgery

## Abstract

**Purpose:**

Survival and treatment intensity in patients with head and neck squamous cell carcinoma (HNSCC) is determined by the presence of lymph node (LN) metastasis, and as a result surgical removal of potentially affected LN remains a mainstay practice. Fluorescence guided surgery (FGS) using targeted optical agents is an expanding field that shows great potential for aiding diagnosis of metastatic LN. Given variations in fluorescence background, a reference standard for regions of interest is necessary for cross patient comparison. The present study aims to determine whether tissue with native target expression can be used as a background to determine metastatic LN in patients with HNSCC infused with anti-epidermal growth factor receptor (EGFR) targeted imaging agents.

**Procedures:**

Twenty-two patients infused with panitumumab-IRDye800 or cetuximab-IRDye800 prior to surgery were included. Fluorescence imaging and analysis was performed on resected LNs (*N* = 843) using the submandibular glands (SMG) and skin as reference standard tissue with known EGFR antigen expression.

**Results:**

Sixteen patients (72.7%) had at least one positive LN on final pathology. The LN to SMG (LN/SMG) and LN to skin (LN/skin) ratios were significantly higher in metastatic LN compared to benign LN (*p* < 0.0001 for both). Using patient-specific ratios to determine an optimal LN/skin cutoff was the most sensitive (95.2%) and directly comparing the LN/skin ratio of all patients to determine a cutoff was the most specific (86.3%).

**Conclusions:**

In HNSCC patients infused with a molecularly targeted fluorescent tracer, endogenous expression of the target antigen can be used as a reference standard to detect LN metastasis. Additionally, the performance of the background in determining metastatic LN can be improved by utilizing patient-specific reference standards.

**Supplementary Information:**

The online version contains supplementary material available at 10.1007/s11307-025-01996-4.

## Introduction

The presence of lymph node (LN) metastasis in head and neck squamous cell carcinoma (HNSCC) greatly reduces survival by up to 50%, highlighting the need to accurately stage patients for proper treatment [1]. Fluorescent guided surgery (FGS) has previously demonstrated the ability to detect metastatic LNs in HNSCC by using IRDye800-labeled panitumumab to target epidermal growth factor receptor (EGFR), which is overexpressed in 80–90% of cases [2–4]. FGS with tumor specific tracers is a growing field; however, there is no gold-standard for objective assessment of fluorescence contrast between tissues. One such area that lacks standardization is the selection of a background tissue, which is necessary to evaluate areas of concern and compare amongst patients. The most common method of doing so involves choosing a background or reference standard that presumably does not express the target and therefore would not have tracer uptake. In the head and neck region, background tissues used in previously published FGS studies have been muscle, fat, unspecified normal or adjacent tissue, and skin [4–6]. Besides skin, all of these areas have low levels of EGFR expression [7, 8].

The use of background that contains endogenous expression of the target receptor has only briefly been explored previously by comparing the fluorescence contrast of primary tumor sections to skin and muscle as backgrounds. Skin proved to be more specific and have better positive and negative predictive values than muscle for cancer identification, supporting the use of EGFR-expressing tissue as a background [6]. However, skin is a composite tissue with variable amounts of dermis and epidermis. In contrast to primary tumors, discrimination of true positive LNs using FGS can be challenging. LNs often have sub-centric metastasis or are embedded in a noisy background and as a result the fluorescence and contrast to surrounding tissue can be challenging to distinguish. Previously, our group published a study showing panitumumab-IRDye800 (pan800) can detect metastatic LNs during pathology processing using fat as a background. The ability of disease-free tissue with native EGFR expression to determine cancerous LNs has not been explored previously.

In addition to skin, the submandibular glands (SMG) present a unique opportunity to serve as a background in head and neck cancer FGS. Paired structures located within level 1b of the neck, these glands natively express EGFR [9, 10]. When level 1b is part of the neck dissection, the SMG is removed and sent to pathology for processing as part of the neck specimen. Even when not removed, the SMG tissue is often within surgical field of view and could potentially serve as a patient control for expected uptake in EGFR-containing tissue. We hypothesize that using tissue with native target expression will be able to detect metastatic LN on resected specimens. The present study aims to compare the mean fluorescence intensity (MFI) of LNs to that of skin and SMG tissue for fluorescence assessment of LN metastasis in patients infused with pan800 or cetuximab-IRDye800.

## Materials and Methods

Data from four prospective, phase 1 clinical trials conducted at Stanford University (SU), Vanderbilt University Medical Center (VUMC), and University of Alabama (UAB) between October 2013 and July 2024 were analyzed (NCT01987375, NCT05945875, NCT02415881, NCT04511078). All studies were reviewed and approved by the respective institutional review boards. Adult patients diagnosed with primary or recurrent HNSCC scheduled to undergo surgery were eligible for inclusion. Written informed consent was obtained from all patients. Trial design and production of study drugs for fluorescence imaging have been described previously [1–5]. Briefly, patients received a pretreatment infusion of up to 100 mg of unlabeled antibody 1 h prior to the study drug, followed by an infusion of cetuximab-IRDye800 (Patients 1–10: 2.5 mg/m^2^, 25 mg/m^2^, or 62.5 mg/m^2^) or panitumumab-IRDye800 (Patients 11–22: 50 mg) 1–5 days prior to surgery. Standard-of-care surgery was performed in all patients. Patients who had fluorescence images of resected LN (total: 843, benign:780, malignant: 63) and SMG or skin tissue specimens were included for analysis in the study.

### Fluorescence Imaging and Histopathology

SMGs were removed as standard practice when performing a level 1b neck dissection. Patients undergoing free-flap reconstruction had a sample of benign skin removed from the flap tissue where possible for comparison with their respective LNs resected. Pathologic processing followed routine standard of care. A closed-field Pearl Impulse or Trilogy imaging system (LI-COR Biosciences Inc.) was used for fluorescence imaging of whole and dissected specimens in cassettes. Manual regions of interest were drawn around each tissue specimen in entirety to determine the MFI using ImageStudio software (V5.2.5, LI-COR Biosciences Inc.). Final pathology on haematoxylin and eosin (H&E) stained sections was determined by a board-certified pathologist and compared to fluorescent imaging.

### LN/Tissue Ratio and Threshold Adjusted Ratio Measurement

The LN/tissue ratio [$LN/tissue ratio= \frac{LN MFI}{Patient Matched Tissue MFI}$] for each individual LN was calculated by dividing the LN MFI by the MFI of the patient-matched SMG (LN/SMG ratio) or skin (LN/skin ratio). Each patient’s mean and standard deviation of the LN/tissue ratio were determined and used to calculate a unique Patient Threshold according to the formula: [$Patient Threshold={Mean}_{Patient}LN/tissue ratio+(0.5\times {Standard Deviation}_{Patient} LN/tissue ratio)$]. The threshold adjusted ratio (TAR) value was determined by taking the LN/tissue ratio of each individual LN and subtracting out the patient-matched unique Patient Threshold determined using SMG or skin as reference tissue, yielding a TAR value for each individual LN as follows: [$TAR=LN/tissue ratio-Patient Threshold$].

### Statistical Analysis

Descriptive statistics and figures were obtained using R Studio. Comparisons of continuous values were performed using Mann–Whitney U-test for two groups. Receiver operating characteristics (ROC) curves were created, and optimal thresholds determined using Youden’s index to provide the sensitivity, specificity, positive predictive value, negative predictive value, and accuracy. DeLong’s test was used for comparison between ROC curves [11]. A *P*-value < 0.05 was considered significant.

## Results

### Patient Characteristics

Twenty-two patients who had a cervical lymphadenectomy (also called selective neck dissection) were included for analysis. Twelve patients (54.4%) were female, and the mean age was 59.5 ± 13.5. Sixteen patients (72.7%) had at least 1 positive LN on final pathology. Subsite and staging information are provided in Table 1.

**Table 1:** Patient demographics

					Staging		Number of Lymph Nodes	
ID	Primary Location	Age	Gender	Prior Radiation	Clinical	Pathologic	Benign	Metastatic
1	Oral Tongue	42	M	No	T2N2b	T1N2b	18	5
2	Cutaneous	50	M	Yes	T3N0	T2N0	41	0
3	Floor of Mouth	77	M	Yes	T4N0	T4aN0	28	0
4	Oral Tongue	40	M	No	T2N1	T2N2b	44	3
5	Hard Palate	84	F	No	T4N0	T2N2b	46	7
6	Oral Tongue	57	M	No	T3N2b	T3N2c	51	11
7	Floor of Mouth	64	F	No	T4N0	T3N0	23	0
8	Buccal Mucosa	69	F	No	T2N0	T2N0	4	0
9	Oral Tongue	38	M	Yes	T4aN1	T4aN2b	16	3
10	Maxillary Gingiva	71	F	No	T4aN0	T2N1	21	2
11	Oral Tongue	73	M	No	T3N1	T3N2b	34	3
12	Oral Tongue	51	F	No	T2N0	T3N1	56	1
13	Oral Tongue	71	F	Yes	T3N0	T2N2b	21	2
14	Oral Tongue	68	F	No	T3N2b	T3N2b	40	5
15	Oral Tongue	59	F	No	T3N2b	T4aN2c	41	5
16	Oral Tongue	57	M	No	T3N0	T4aN3b	53	6
17	Oral Tongue	70	M	No	T3N0	T4aN3b	40	2
18	Oral Tongue	53	F	No	T2N1	T3N1	56	1
19	Base of Tongue	68	M	Yes	T3N1	T4N1	11	5
20	Oral Tongue	35	F	No	T1N1	T2N2b	44	2
21	Oral Tongue	49	F	No	T3N2c	T4aN0	29	0
22	Oral Tongue	64	F	No	T2N0	T4aN0	63	0

Abbreviations: M, male; F, female

### LN/SMG Ratio

**Fig. 1:**
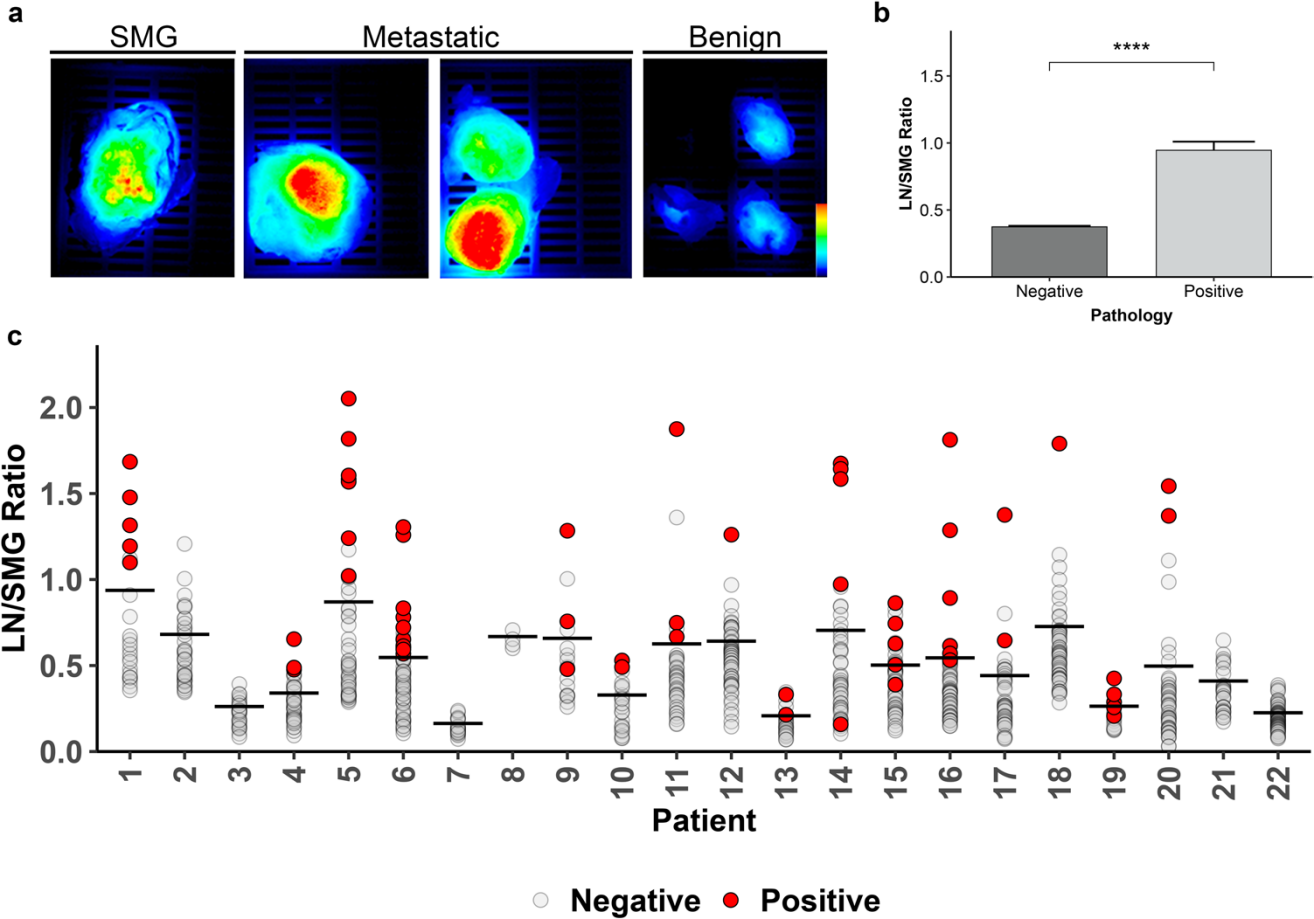
LN/SMG ratio using SMG as reference (**a**) Representative closed-field fluorescence imaging of specimens in cassettes from patient 20 with all images set to the same scale. (**b**) The overall mean of LN/SMG ratio for benign and malignant lymph nodes. Error bars represent the standard error of measurement. ****p < 0.0001. (**c**) The LN/SMG ratio (mean indicated by horizontal line) of all lymph nodes separated by patient. LN: lymph node; SMG: submandibular gland

For patient-matched SMG analysis, there were a total of 843 LN included of which 63 (7.4%) had malignancy present on final pathology. Representative fluorescence images of resected SMG, metastatic LN, and benign LN are provided in Fig. 1a. The mean LN/SMG ratio of malignant LNs (0.95, standard error (SE) = 0.06) was more than twice that of benign LN (0.37, SE = 0.008, *p* < 0.0001; Fig. 1b) across the patient cohort. Figure 1c indicates the individual LN/SMG ratios and mean values for each patient.

### LN/Skin Ratio

**Fig. 2:**
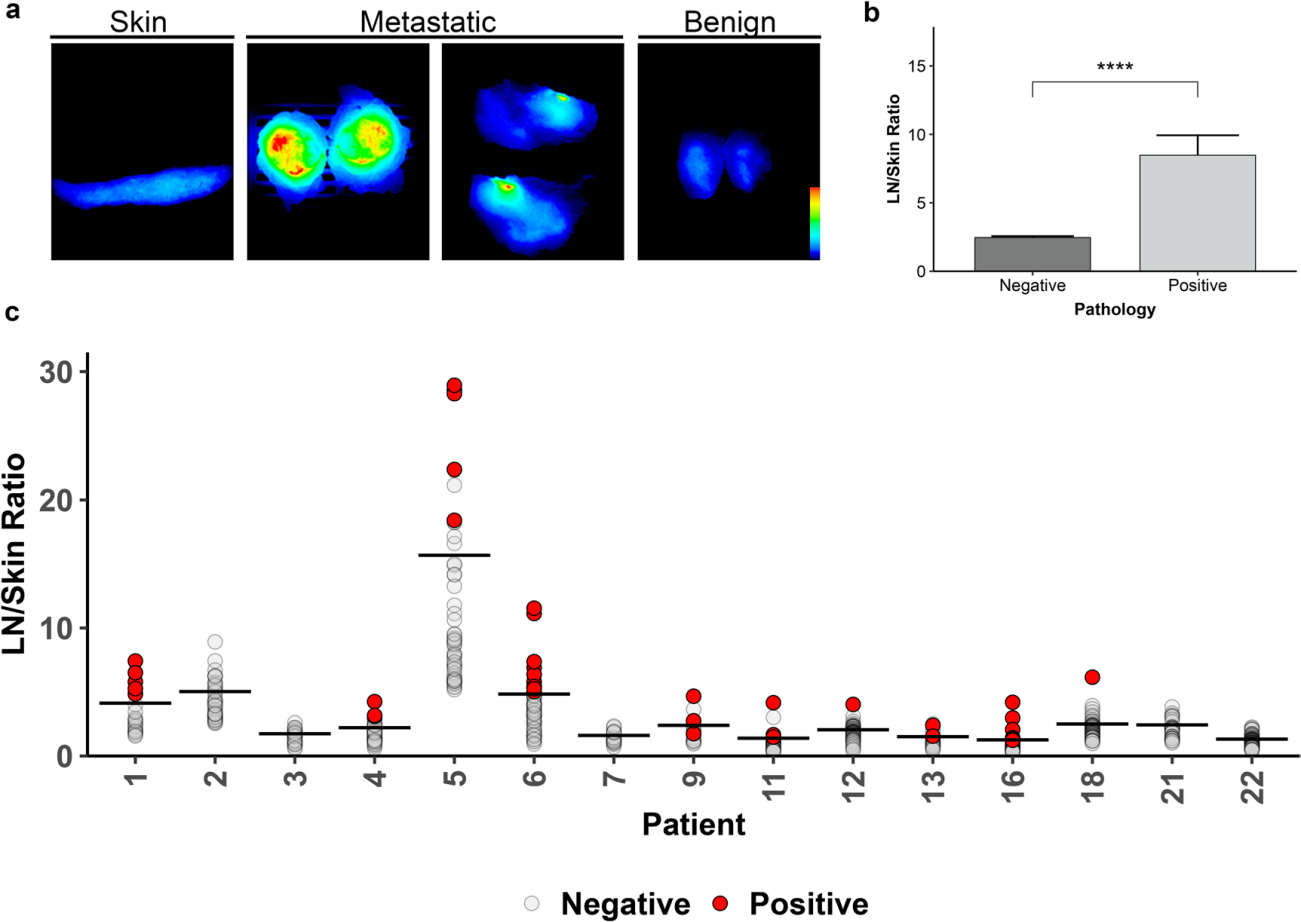
LN/tissue ratio using skin as a reference. (**a**) Representative closed-field fluorescence imaging of specimens from patient 11 with all images set to the same scale. (**b**) The combined mean LN/skin ratio for benign and metastatic lymph nodes. Error bars represent the standard error of measurement. ****p < 0.0001. (**c**) The LN/skin ratio (mean indicated by horizontal line) of all lymph nodes separated by patient. LN: lymph node

Fifteen patients had skin available for patient-matched analysis of LN fluorescence. Fluorescence in skin was low relative to the fluorescence observed in metastatic LN from these patients (Fig. 2a). A total of 621 LNs were included, 42 (8.4%) of which were positive for malignancy. Malignant LNs had a mean LN/skin ratio (8.47, SE = 1.46) that was three-times greater than that of benign LN (2.46, SE = 1.46, *p* < 0.0001; Fig. 2b). Individual LN and mean patient LN/skin ratios are indicated in Fig. 2c.

### ROC Analysis

**Fig. 3:**
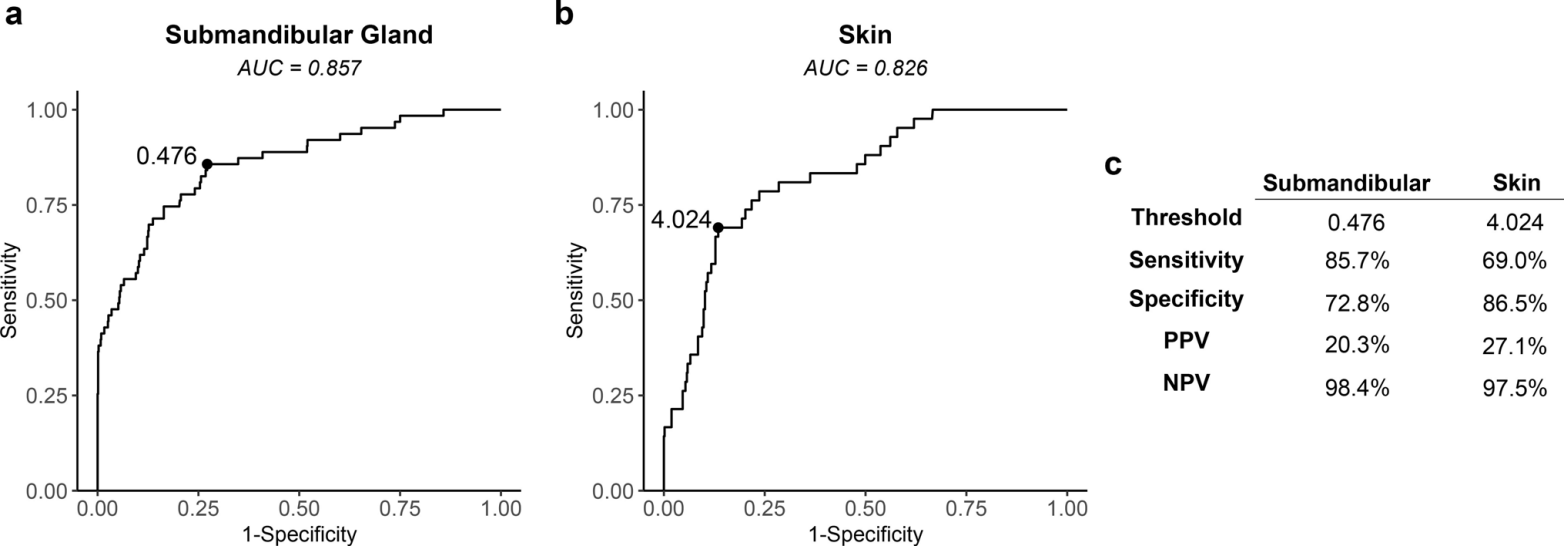
Performance of both LN/tissue ratios. (**a**, **b**) Receiver operating curves for the LN/SMG ratio (left) and LN/skin ratio (right). (**c**) The threshold for each LN/tissue ratio determined by Youden’s index and the sensitivity, specificity, positive predictive value (PPV) and negative predictive value (NPV) for each. LN: lymph node; SMG: submandibular gland

ROC analysis using LN/SMG ratios to distinguish malignant from benign nodes (Fig. 3a) showed an area under the curve (AUC) of 0.857 (95% CI, 0.80–0.91). The AUC of the ROC using LN/skin ratios to distinguish malignant from benign LNs (Fig. 3b) was 0.826 (95% CI, 0.76–0.89). The optimal LN/SMG ratio cutoff value determined from Youden’s index was 0.476, yielding a sensitivity of 85.7% (95% CI, 0.78–0.94) and specificity of 72.8% (95% CI, 0.70–0.76; Fig. 3c). The best LN/skin ratio cutoff value determined from Youden’s index was 4.024, which provided a sensitivity of 69.0% (95% CI, 0.55–0.83) and a specificity of 86.5% (95% CI, 0.84–0.89; Fig. 3c). Both of the ratios had better AUC compared to raw MFI alone (Supplementary Fig. 1).

### Threshold Adjusted Ratio (TAR) Measurement

**Fig. 4:**
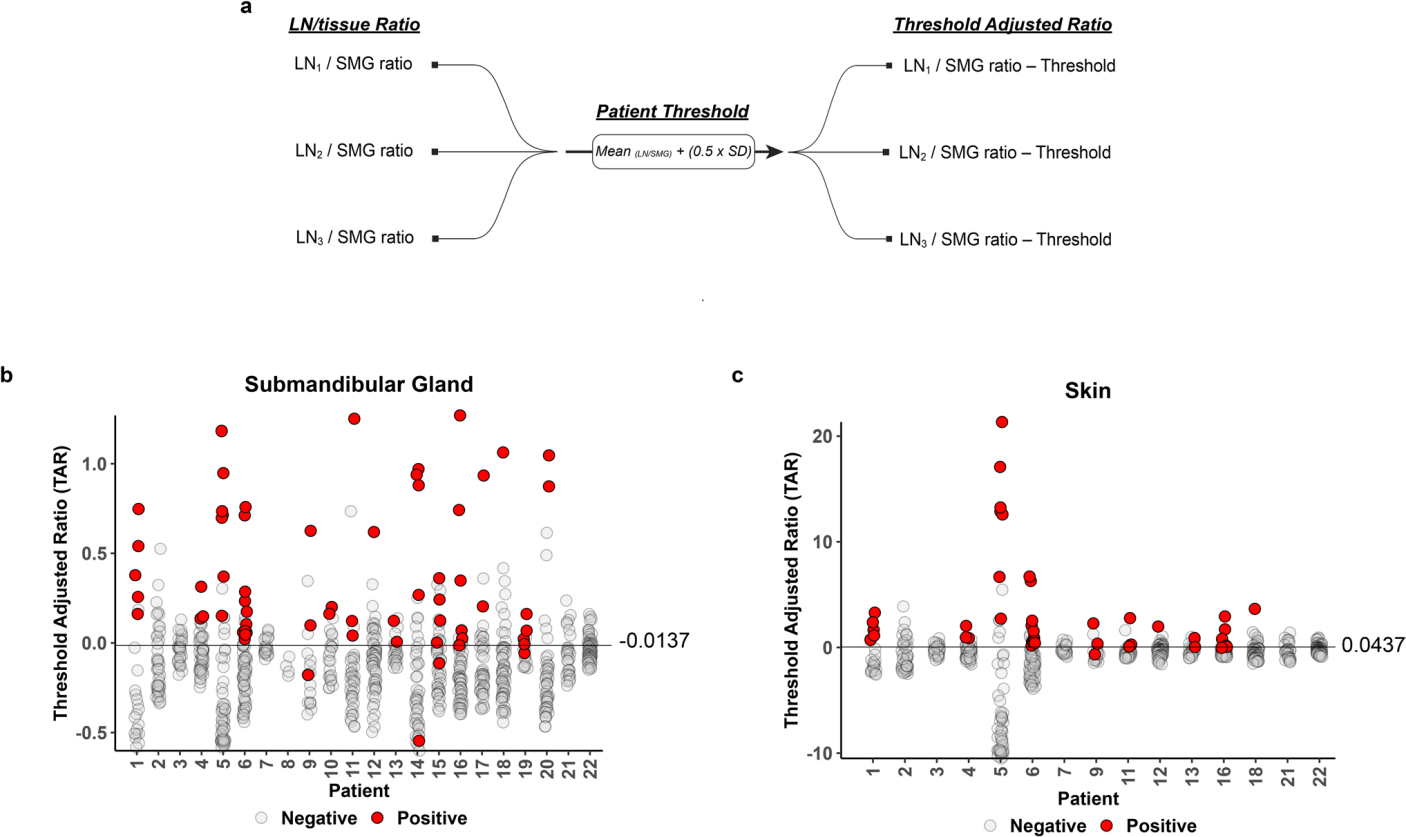
Threshold Adjusted Ratios of all lymph nodes. **(a**) Patients had individual thresholds calculated by taking their mean LN/tissue ratio plus one-half the standard devidation. A threshold adjusted ratio (TAR) was calculated by the LN/tissue ratio subtracted by the threshold for each lymph node by patient. (**b**, **c**) Each lymph node TAR plotted by submandibular gland (left) and skin (right). The line on each plot represents the respective uniform cutoff TAR

**Fig. 5:**
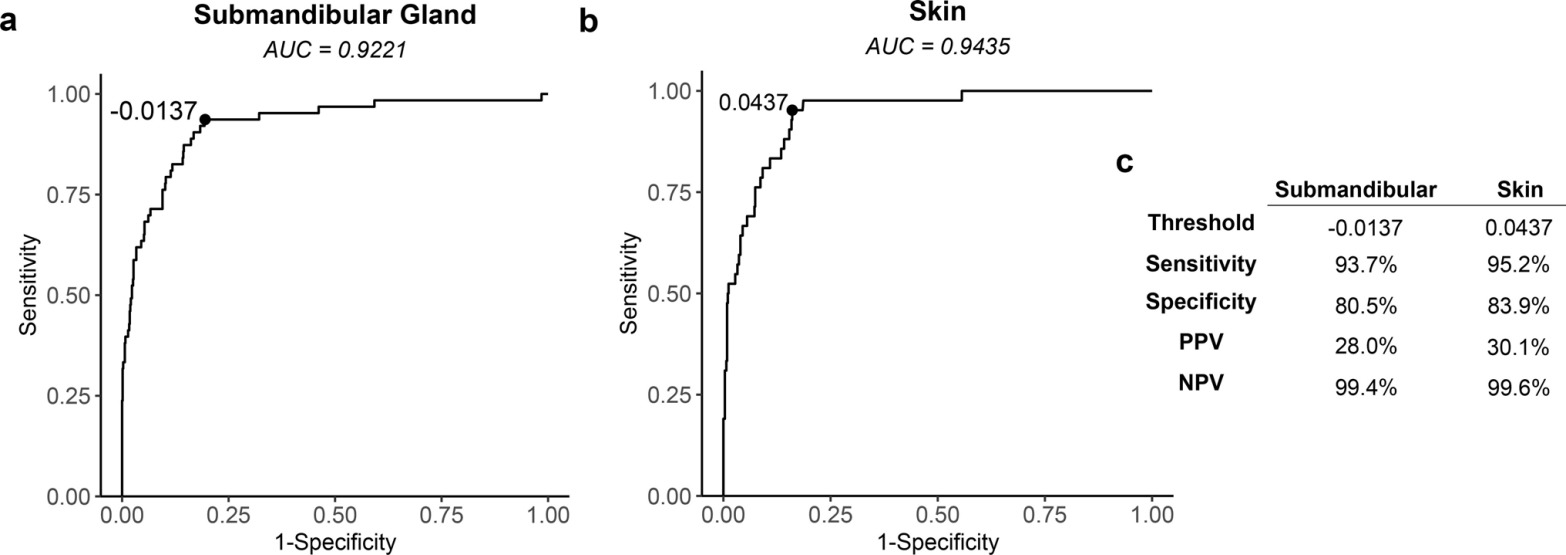
The performance of the TAR_SMG_ and TAR_skin_. (**a**, **b**) Receiver operating curves for the TAR_SMG_ (left) and TAR_skin_ (right). (**c**) The threshold for each TAR determined by Youden’s index shows improved sensitivity and negative predictive value (NPV). TAR: threshold adjusted ratio; LN: lymph node; SMG: submandibular gland; PPV: positive predictive value

The moderate accuracy of LN/tissue ratios to distinguish malignant from benign LN prompted exploration of alternative fluorescence metrics that could provide greater diagnostic accuracy. After considering the wide variation of LN/tissue ratios both within and across patients, we derived a patient-specific threshold from the LN/tissue ratio and subtracted this threshold value from the LN/tissue ratio determined for each patient-matched LN (Fig. 4a). Applying this threshold adjusted ratio (TAR) approach to each LN approximately normalized the resulting mean values for each patient when using the SMG (TAR_SMG_) (Fig. 4b) or skin (TAR_skin_) (Fig. 4c) as reference tissue. ROC analyses of the TAR_SMG_ (Fig. 5a) and TAR_skin_ (Fig. 5b) values provided an AUC of 0.922 (95% CI, 0.88–0.96) and 0.944 (95% CI, 0.91–0.97), respectively, to differentiate malignant from benign LN. Both AUCs were greater than the threshold adjusted MFI performance (Supplementary Fig. 1). The improved AUC of the TAR_SMG_ was significant when compared to the AUC for the LN/SMG ratio (*p* = 0.001). Additionally, the greater AUC for the TAR_skin_ was significant when compared to the AUC for the LN/skin ratio (*p* < 0.0001). Optimal TAR_SMG_ and TAR_skin_ cutoff values determined by Youden’s index (−0.0137 and 0.0437, respectively) provided sensitivities of 93.7% (95% CI, 0.87–0.98) and 95.2% (95% CI, 0.88–1.00), respectively, and specificities of 80.5% (95% CI, 0.78–0.83) and 83.9% (95% CI, 0.80–0.87), respectively (Fig. 5c). Overall, the TAR_SMG_ and TAR_skin_ results showed superior diagnostic value to distinguish malignant from benign LN relative to the LN/tissue ratios (Fig. 3c) when using SMG and skin as reference tissues for fluorescence assessment.

## Discussion

Cancer-specific optical agents such as fluorescently labeled monoclonal antibodies are actively under investigation for solid tumors, but the primary focus of these investigations have been the primary tumor and the LN metastasis has been largely unexamined [12]. In this study, we analyzed 22 patients with HNSCC who underwent infusion with an anti-EGFR imaging agent followed by FGS and identified a novel approach using LN/tissue ratios to objectively assess fluorescent signal in metastatic LNs.

Head and neck tumors frequently produce metastasis in cervical LN, and the removal of these nodes is not without morbidity like shoulder dysfunction, lymphedema, impaired cosmesis, among others. This indicates a clinical need to develop novel approaches to identify metastatic LNs for selective resection in the most minimally invasive manner. Our quantitative method of normalizing LN MFI by the respective patient’s SMG and skin MFI values produced ratio (LN/tissue) thresholds with either favorable sensitivity or specificity for predicting the presence of cancer in the tissue tested. To further improve accuracy, we calculated a patient-specific threshold by assessing the mean plus one-half standard deviation of the LN/tissue ratios determined from a patient’s individual lymph nodes. By subtracting this patient-specific threshold from the individual LN/tissue ratio per node, we were able to generate a threshold adjusted ratio (TAR) to allow comparison between patients. While this method showed > 93% sensitivity in identifying malignant LN, inherent variations of EGFR expression and antibody retention across tissues confound the specificity. However, considering the consequences of a false negative during cancer localization in the surgical setting, the optimal threshold should be weighted towards the greatest sensitivity to improve detection of true positive tissues.

The results of this study are in agreement with prior work using EGFR-targeted fluorescence imaging to detect malignant LNs, as well as a previous report of a semi-quantitative threshold for delineating primary tumor during FGS in HNSCC [3, 4, 6, 13]. In our study, skin showed superiority to the SMG as a background tissue for determining metastasis. The TAR_skin_ cutoff had the highest sensitivity, and the LN/skin ratio had the highest specificity overall. Compared to the prior study using fibro-adipose tissue as background, our method showed higher sensitivity using both the TAR_SMG_ and TAR_skin_ cutoff values and marginally better specificity using the LN/skin cutoff value [4]. Although there were 4 false negative lymph nodes using the TAR_SMG_ and 2 false negative lymph nodes using the TAR_skin_ cutoff, these results are similar to the previous studies [3, 4]. Only one patient had a metastasis missed by both the TAR_SMG_ and TAR_skin_ cutoff, however, multiple other metastatic lymph nodes were present and correctly identified in this patient. One hypothesis for the false negatives is size of the metastasis, as this can affect the amount of tracer localization with micrometastasis having less accumulation due to less target receptor presence.

One limitation of this study is the retrospective design and ability to only include patients that had skin or SMG samples for review. Although each patient had many nodes to include, the metastatic lymph nodes accounted for less than 10% of total LNs. Additionally, analysis was limited to post-resection, closed-field imaging of specimens and not applicable to real-time surgery currently. Despite these limitations, we were able to identify a novel approach using ratios from tissues known to contain the targeted antigen (EGFR in this case) to serve as a needed tool for standardizing LN evaluation in patients who undergo FGS. This tool would be ideal for improving the pathology workflow by allowing the pathologist to preselect lymph nodes for evaluation based on their comparison to the patient’s own tissue. These ratiometric parameters could be applied to a wide variety of tumor types and anatomic locations depending on the targeted tracer used, further improving oncologic surgery.

## Conclusions

Results of the current study demonstrate how benign tissue with physiologic target expression in FGS can be used to identify lymph nodes concerning for metastatic disease. Further studies evaluating the clinical feasibility and accuracy of this method in open-field surgery would be beneficial.

## Supplementary Information

Below is the link to the electronic supplementary material.

Supplementary file1 (DOCX 126 KB)

## Data Availability

All research data and computer codes are available from the corresponding author upon request.

## References

[1] Gourin CG, Conger BT, Porubsky ES, Sheils WC, Bilodeau PA, Coleman TA (2008) The effect of occult nodal metastases on survival and regional control in patients with head and neck squamous cell carcinoma. Laryngoscope 118(7):1191–1194. 10.1097/MLG.0b013e31816e2eb7 18391764 10.1097/MLG.0b013e31816e2eb7

[2] Kalyankrishna S, Grandis JR (2006) Epidermal growth factor receptor biology in head and neck cancer. J Clin Oncol 24(17):2666–2672. 10.1200/JCO.2005.04.8306 16763281 10.1200/JCO.2005.04.8306

[3] Krishnan G, van den Berg NS, Nishio N, Juniper G, Pei J, Zhou Q, Lu G, Lee YJ, Ramos K, Iagaru AH et al (2021) Metastatic and sentinel lymph node mapping using intravenously delivered Panitumumab-IRDye800CW. Theranostics. 11(15):7188–7198. https://www.ncbi.nlm.nih.gov/pubmed/34158844. 10.7150/thno.55389 10.7150/thno.55389 PMC8210603 34158844

[4] Nishio N, van den Berg NS, van Keulen S, Martin BA, Fakurnejad S, Teraphongphom N, Chirita SU, Oberhelman NJ, Lu G, Horton CE et al (2019) Optical molecular imaging can differentiate metastatic from benign lymph nodes in head and neck cancer. Nat Commun 10(1):5044. https://www.ncbi.nlm.nih.gov/pubmed/31695030. 10.1038/s41467-019-13076-7 10.1038/s41467-019-13076-7 PMC6834597 31695030

[5] de Wit JG, Vonk J, Voskuil FJ, de Visscher SAHJ, Schepman K-P, Hooghiemstra WTR, Linssen MD, Elias SG, Halmos GB, Plaat BEC et al (2023) EGFR-targeted fluorescence molecular imaging for intraoperative margin assessment in oral cancer patients: a phase II trial. Nat Commun 14(1):4952. 10.1038/s41467-023-40324-8 37587149 10.1038/s41467-023-40324-8 PMC10432510

[6] Warram JM, de Boer E, Moore LS, Schmalbach CE, Withrow KP, Carroll WR, Richman JS, Morlandt AB, Brandwein-Gensler M, Rosenthal EL (2015) A ratiometric threshold for determining presence of cancer during fluorescence-guided surgery. J Surg Oncol 112(1):2–8. https://www.ncbi.nlm.nih.gov/pubmed/26074273. 10.1002/jso.23946 10.1002/jso.23946 PMC4510011 26074273

[7] Fagerberg L, Hallström BM, Oksvold P, Kampf C, Djureinovic D, Odeberg J, Habuka M, Tahmasebpoor S, Danielsson A, Edlund K et al (2014) Analysis of the human tissue-specific expression by genome-wide integration of transcriptomics and antibody-based proteomics. Mol Cell Proteomics 13(2):397–406. 10.1074/mcp.M113.035600 24309898 10.1074/mcp.M113.035600 PMC3916642

[8] Edqvist P-HD, Fagerberg L, Hallström BM, Danielsson A, Edlund K, Uhlén M, Pontén F (2015) Expression of human skin-specific genes defined by transcriptomics and antibody-based profiling. J Histochem Cytochem 63(2):129–141. 10.1369/0022155414562646 25411189 10.1369/0022155414562646 PMC4305515

[9] Dubiel B, Mytar B, Kielar B, Tarnawski A, Zembala M, Stachura J (1992) Immunolocalization of epidermal growth factor (EGF) in human salivary glands detected with the new monoclonal antibody. Patol Pol 43(2):55–57 1296177

[10] Piludu M, Lantini MS, Isola M, Puxeddu R, Cossu M (2003) Localisation of epidermal growth factor receptor in mucous cells of human salivary glands. Eur J Morphol 41(2):107–109. 10.1080/09243860412331282219 15621865 10.1080/09243860412331282219

[11] DeLong ER, DeLong DM, Clarke-Pearson DL (1988) Comparing the areas under two or more correlated receiver operating characteristic curves: a nonparametric approach. Biometrics 44(3):837. 10.2307/2531595. Accessed 2024 Dec 25 3203132

[12] Bou-Samra P, Muhammad N, Chang A, Karsalia R, Azari F, Kennedy G, Stummer W, Tanyi J, Martin L, Vahrmeijer A et al (2023) Intraoperative molecular imaging: 3rd biennial clinical trials update. J Biomedical Optics 28 (05). 10.1117/1.JBO.28.5.050901 10.1117/1.JBO.28.5.050901 PMC10182831 37193364

[13] Rosenthal EL, Moore LS, Tipirneni K, de Boer E, Stevens TM, Hartman YE, Carroll WR, Zinn KR, Warram JM (2017) Sensitivity and specificity of cetuximab-IRDye800CW to identify regional metastatic disease in head and neck cancer. Clin Cancer Res 23(16):4744–4752. 10.1158/1078-0432.CCR-16-2968 28446503 10.1158/1078-0432.CCR-16-2968 PMC5595145

