# Supplementary material for: Background Tissue with Native Target Expression Can Determine Presence of Nodal Metastasis in Head and Neck Squamous Cell Carcinoma Patients Infused with Targeted Fluorescent Tracers
Source: Mol Imaging Biol. 2025 Mar 18;27(3):333–40. doi: 10.1007/s11307-025-01996-4 (PMC12162778; doi:10.1007/s11307-025-01996-4)
Supplement: Supplementary file 1 — Supplementary file1 (DOCX 126 KB) [file 11307_2025_1996_MOESM1_ESM.docx]

**Electronic Supplementary Material**

**Background Tissue with Native Target Expression Can Determine Presence of Nodal Metastasis in Head and Neck Squamous Cell Carcinoma Patients Infused with Targeted Fluorescent Tracers**

**Journal: Molecular Imaging and Biology**

Nicole Meeks^1^, Sherin James^2^, Giri Krishnan^3^, Akhilesh Wodeyar^2^, Hidenori Tanaka^1^, Benjamin B. Kasten^2^, Yu-Jin Lee^4^, Marisa E. Hom^1^, Eben L. Rosenthal^1^, Jason M. Warram^2^

1. Department of Otolaryngology-Head and Neck Surgery, Vanderbilt University Medical Center, Nashville, TN, USA
2. Department of Otolaryngology, The University of Alabama at Birmingham, Birmingham, AL, USA
3. Department of Otolaryngology, The University of Adelaide, Adelaide, South Australia, Australia
4. Department of Otolaryngology-Head and Neck Surgery, Stanford University School of Medicine, Stanford, CA, USA

**Corresponding author:**

Jason M. Warram

1670 University Blvd, Volker Hall G082

Birmingham, AL, USA, 35294

Tel: +1-(205)-996-5000

Email: [mojack@uab.edu](mailto:mojack@uab.edu)

**Materials and Methods**

Determination of the mean fluorescence intensity (MFI) for each lymph node (LN) occurred as described in the methods. For the MFI threshold adjusted ratio (TAR), each patient had an individual threshold calculated as: $Patient Threshold={Mean}_{Patient}MFI+\left( 0.5 \times{Standard Deviation}_{Patient} MFI \right)$. The threshold adjusted ratio (TAR) value was determined similarly to the TAR_SMG_ and TAR_skin_ by taking the MFI of each individual LN and subtracting out the patient-matched unique Patient Threshold.

**Results**

The area under the curve (AUC) for the raw MFI values was 0.736 (95% CI: 0.66-0.81; Supplemental Figure 1). The optimal MFI cutoff determined by Youden’s was 0.121 with a sensitivity of 55.5% (95% CI: 42.9-68.3%) and specificity of 81.9% (95% CI: 79.3-84.6%). For the TAR­_MFI­­_, AUC was 0.914 (95% CI: 0.87-0.96) with an optimal threshold of 0.00005, yielding a sensitivity of 90.5% (95% CI: 82.5-96.8%) and specificity of 83.0% (95% CI: 80.3-85.4%).


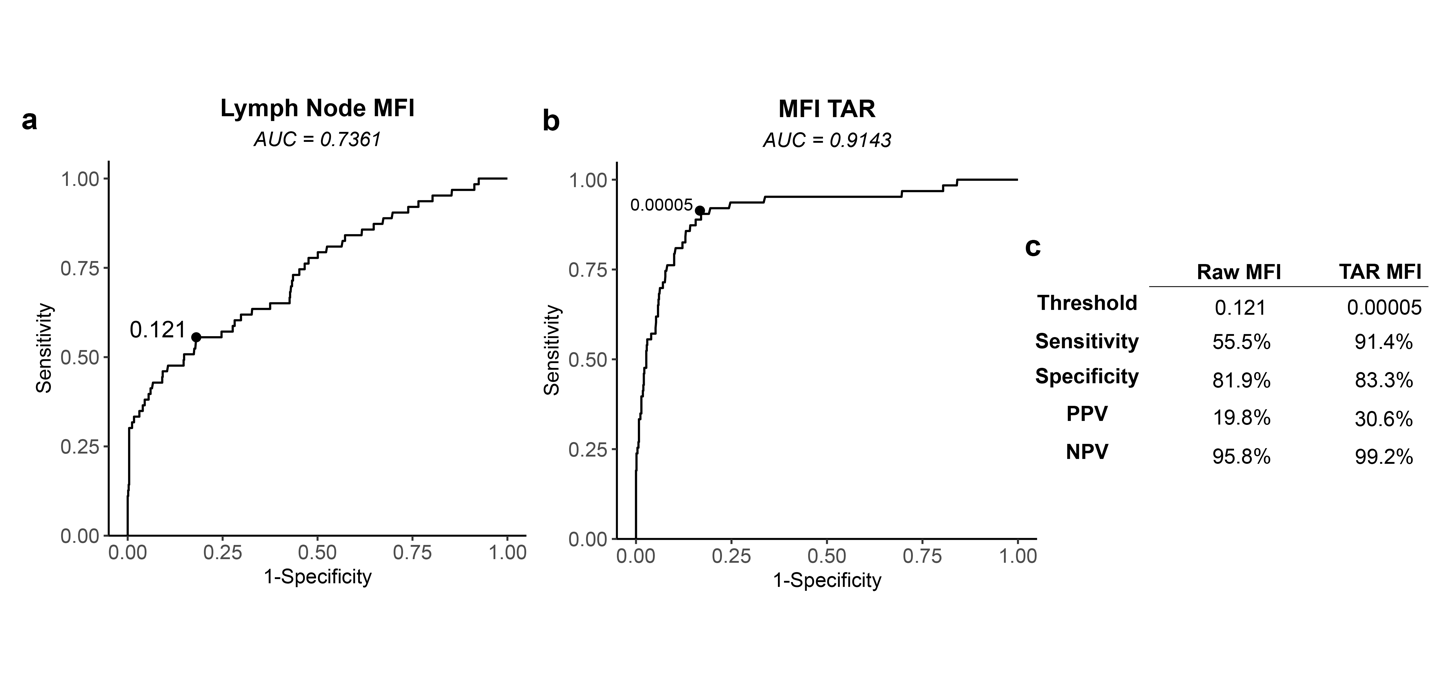


**Supplementary Figure 1.** Performance of MFI alone and TAR_MFI_ **(a)** Receiver operating curves for MFI alone. **(b)** Receiver operating curve for TAR_MFI_. **(c)** The threshold for the MFI alone and TAR_MFI_ determined by Youden’s index. MFI: mean fluorescence intensity; TAR: threshold adjusted ratio; PPV: positive predictive value; NPV: negative predictive value.
